# The Potential of Asiatic Acid in the Reversion of Cyclophosphamide-Induced Hemorrhagic Cystitis in Rats

**DOI:** 10.3390/ijms22115853

**Published:** 2021-05-29

**Authors:** Andrzej Wróbel, Łukasz Zapała, Tomasz Kluz, Artur Rogowski, Marcin Misiek, Kajetan Juszczak, Jacek Sieńko, Daniela Gold, Klaudia Stangel-Wójcikiewicz, Ewa Poleszak, Piotr Radziszewski

**Affiliations:** 1 Second Department of Gynecology, Medical University of Lublin, Jaczewskiego 8, 20-090 Lublin, Poland; 2 Clinic of General, Oncological and Functional Urology, Medical University of Warsaw, Lindleya 4, 02-005 Warsaw, Poland; pradziszewski@wum.edu.pl; 3 Department of Gynecology and Obstetrics, Institute of Medical Sciences, Medical College of Rzeszow University, 35-310 Rzeszow, Poland; jtkluz@interia.pl; 4 Faculty of Medicine, Collegium Medicum, Cardinal Stefan Wyszynski University in Warsaw, 01-938 Warsaw, Poland; artur.rogowski@imid.med.pl; 5 Department of Obstetrics and Gynecology, Mother and Child Institute, 01-211 Warsaw, Poland; 6 Department of Gynecologic Oncology, Holy Cross Cancer Center, 25-377 Kielce, Poland; marcin.misiek@icloud.com; 7 Chair of Urology and Andrology, Collegium Medicum in Bydgoszcz, Nicolaus Copernicus University in Torun, Skłodowskiej-Curie 9, 85-094 Bydgoszcz, Poland; kaj.juszczak@gmail.com; 8 Second Department of Obstetrics and Gynecology, Medical University of Warsaw, 02-091 Warszawa, Poland; jacek.sienko@wum.edu.pl; 9 Department of Obstetrics and Gynecology, Medical University of Graz, Auenbruggerplatz, 8036 Graz, Austria; daniela.gold@medunigraz.at; 10 Department of Gynecology and Oncology, Jagiellonian University Medical College, 31-501 Cracow, Poland; ksw@cm-uj.krakow.pl; 11 Chair and Department of Applied and Social Pharmacy, Laboratory of Preclinical Testing, Medical University of Lublin, 1 Chodźki Street, 20-093 Lublin, Poland; ewapoleszak@umlub.pl

**Keywords:** asiatic acid, cyclophosphamide, hemorrhagic cystitis

## Abstract

The purpose of this study was to determine if asiatic acid may act efficiently in the model of cyclophosphamide (CYP)-induced cystitis in rats. We performed experiments after administration of CYP (single dose 200 mg/kg, intraperitoneally), asiatic acid (30 mg/kg/day for 14 consecutive days, by oral gavage), or CYP plus asiatic acid, during which conscious cystometry, measurements of urothelium thickness and bladder edema, as well as selected biomarkers analyses were conducted. In rats that received asiatic acid together with CYP, a drop in bladder basal pressure, detrusor overactivity index, non-voiding contraction amplitude, non-voiding contraction frequency, and the area under the pressure curve were observed, when compared to the CYP group. Furthermore, a significant increase in threshold pressure, voided volume, intercontraction interval, bladder compliance, and volume threshold to elicit NVC were found in that group accordingly. Administration of the asiatic acid successfully restored concentrations of biomarkers both in bladder urothelium (BDNF, CGRP, OCT-3, IL-1β, IL-6, NGF, nitrotyrosine, malondialdehyde, TNF-α, SV2A, SNAP23, SNAP25, PAC-1, ORM1, occludin, IGFBP-3, HB-EGF, T–H protein, Z01, and HPX) and detrusor muscle (Rho kinase and VAChT) in CYP-treated rats. Finally, asiatic acid significantly decreased urothelium thickness and bladder oedema. Asiatic acid proved to be a potent and effective drug in the rat model of CYP-induced cystitis.

## 1. Introduction

Natural products have already attracted researchers’ attention for their use in many medical applications. One of the new candidates is *Centella asiatica*, which represents a medicinal herb that may be a source of several natural agents, i.e., pentacyclic triterpenoid saponins, inclusively known as centelloids [1]. An example of these chemical substances is asiatic acid (AA), an aglycone form of asiaticoside [2,3], also found in other plants [4]. The activity of AA and its potential role in the treatment of various chronic disorders are due to its pharmacological properties, low toxicity, and commercial availability [5]. When analyzing the compound closely, it occurs that it exhibits a wide spectrum of biological activities, markedly anti-inflammatory and wound healing [6], as well as neuroprotective [7] or neuromodulatory properties [8]. Thus, among its specific actions there are the modulation of oxidative stress and inflammatory cytokine production [9].

As a consequence, one of the possible applications of AA would be devoted to the prevention of inflammatory disorders. AA possesses wide properties of activation of various receptors, i.e., PPAR-γ, GABAA, and GABAB receptors [4]. Reverse action was observed in case of angiotensin (AT1), endothelin 1 (ET1), or toll-like receptors (TLR-4). Furthermore, due to its broad spectrum of properties, AA suppresses α-glucosidase, leukotriene C4 synthase, HMG-CoA-reductases, eNOS/iNOS, PARP, COX-2, and activates collagen-1 or acetylcholine synthesis [4,6]. Thus, it is hard to pinpoint the unique or single molecular target or mechanism, rather than multimodal effects, which in turn leads to the modulation of many molecular targets by altering their gene expression by AA or signaling pathways.

It is evident, though, that there is very limited data on its effects in other specific diseases, including cyclophosphamide-induced cystitis. Cyclophosphamide (CYP), which is a commonly used drug owing to oncological and immunosuppressive indications, can promote exaggerated voiding frequency and chronic pelvic pain [10]. The pathological mechanism of its actions is dedicated to accumulation of acrolein in urothelium, which imposes reactive oxygen species production and causes direct damage to the bladder wall, leading to hemorrhagic cystitis [11]. The optimal treatment in that condition remains undetermined: mesna is the only official drug for CYP-induced bladder damage, though the majority of CYP side effects persist even in the presence of the medication [12]. On the other hand, many papers suggest that natural products may exhibit antioxidant activity, which in turn occurs to be beneficial in CYP-induced urotoxicity [13]. Recent ongoing preclinical studies are focused on the assessment of new pharmacological modalities, e.g., activation of cannabinoid receptors in this setting [14]. Finally, AA was found to possess both anti-inflammatory and antioxidant effects in animals with CYP-induced bladder disorders e.g., via the inhibition of NLRP3 inflammasome activation and the NF κB pathway [15]. In our previous paper [16], in the retinyl acetate-induced model of detrusor overactivity we concluded that AA may be a candidate for its use in the prevention and treatment of overactive bladder and proved that asiatic acid can be implemented in the prevention of conditions accompanied by detrusor overactivity.

The aim of the present study was to determine if AA would reverse the CYP-induced changes in several cystometric and inflammatory parameters, indicating the development of bladder inflammation and bladder overactivity. On these grounds, AA may become a rational strategy in the management of CYP-induced hemorrhagic cystitis.

## 2. Results

### 2.1. The Results of Pretreatment with Asiatic Acid on Cyclophosphamide-Induced Changes in Cystometric Parameters

A cyclophosphamide injection led to a significant increase in the following parameters in conscious cytometry: basal pressure (BP), detrusor overactivity index (DOI), non-voiding contraction amplitude (ANVC), non-voiding contraction frequency (FNVC), and the area under the pressure curve (cm H_2_O/s) (Table 1). On the contrary, threshold pressure (TP), voided volume (VV), intercontraction interval (ICI), bladder compliance (BC), and volume threshold to elicit NVC (VTNC) were markedly decreased when compared to the control group. Importantly, in rats that received asiatic acid together with cyclophosphamide a major drop in BP, DOI, ANVC, FNVC, and AUC was observed when compared to the CYP group. Furthermore, administration of asiatic acid to animals that were CYP-injected resulted insignificant increase in TP, VV, ICI, BC, and VTNC, when collated with the CYP group.

### 2.2. The Influence of Pretreatment with Asiatic Acid on Cyclophosphamide-Induced Changes in Bladder Urothelium and Detrusor Biomarkers

**Figure 1:**
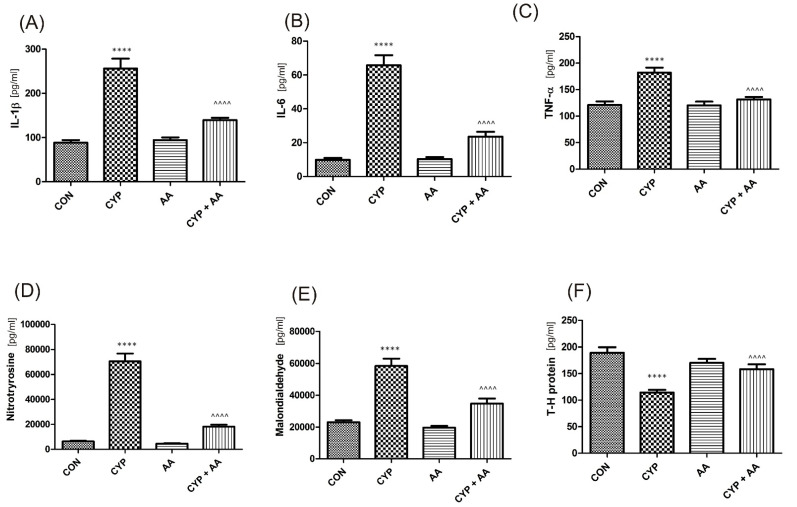
The effect of the two-week administration of asiatic acid (30 mg/kg/day for 14 days by oral gavage) on biomarkers in urothelium or in bladder detrusor muscle: (**A**) interleukin 1-β (IL-1β), (**B**) interleukin 6 (IL-6), (**C**) tumor necrosis factor alpha (TNF-α), (**D**) 3-nitrotyrosine (NIT), (**E**) malondialdehyde (MAL), and (**F**) Tamm–Horsfall protein (T–H protein) in animals that were administered with a single injection of cyclophosphamide (CYP, 200 mg/kg, intraperitoneally). All results are presented as the mean ± SEM (n = 15 rats per group). The obtained data were assessed by the one-way ANOVA followed by Tukey’s post hoc test. * when significantly different from the control group. ^ when significantly different from the CYP group. **** or ^^^^ *p* < 0.0001. One-way ANOVA: for IL-1β F(3.56) = 41, *p* < 0.0001, for IL-6 F(3.56) = 59, *p* < 0.0001, for TNF-α F(3.56) = 17, *p* < 0.0001, for NIT F(3.56) = 91, *p* < 0.0001, for MAL F(3.56) = 36, *p* < 0.0001, and for T–H protein F(3.56) = 15, *p* < 0.0001. AA: asiatic acid; CON: control; CYP: cyclophosphamide.

**Figure 2:**
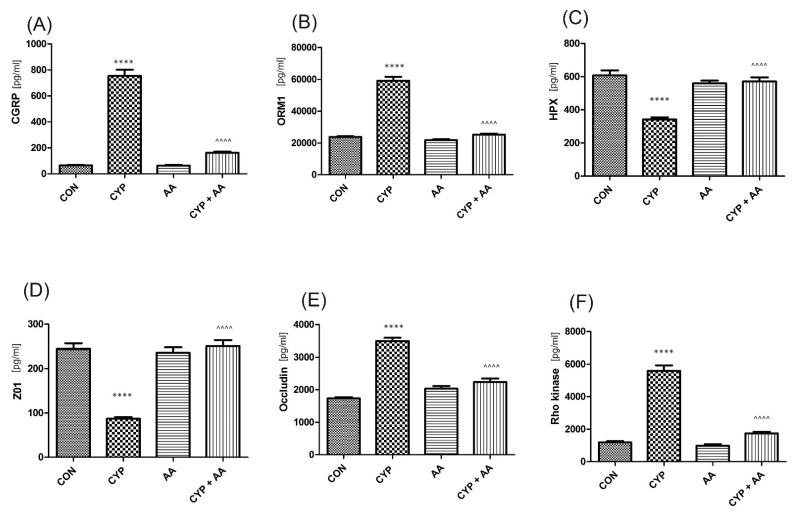
The effect of the two-week administration of asiatic acid (30 mg/kg/day for 14 days by oral gavage) on biomarkers in urothelium or in bladder detrusor muscle: (**A**) calcitonin gene related peptide (CGRP), (**B**) orosomucoid-1 (ORM1), (**C**) hemopexin (HPX), (**D**) tight junction protein 1 (ZO1), (**E**) occludin (OCC), and (**F**) Rho kinase (ROCK1) in animals that were administered with a single injection of cyclophosphamide (CYP, 200 mg/kg, intraperitoneally). All results are presented as the mean ± SEM (n = 15 rats per group). The obtained data were assessed by the one-way ANOVA followed by Tukey’s post hoc test. * when significantly different from the control group. ^ when significantly different from the CYP group. **** or ^^^^ *p* < 0.0001. One-way ANOVA: for CGRP F(3.56) = 185, *p* < 0.0001, for ORM1 F(3.56) = 176, *p* < 0.0001, for HPX F(3.56) = 29, *p* <0.0001, for ZO1 F(3.56) = 49, *p* < 0.0001, for OCC F(3.56) = 82, *p* < 0.0001, and for ROCK1 F(3.56) = 131, *p* < 0.0001. AA: asiatic acid; CON: control; CYP: cyclophosphamide.

**Figure 3:**
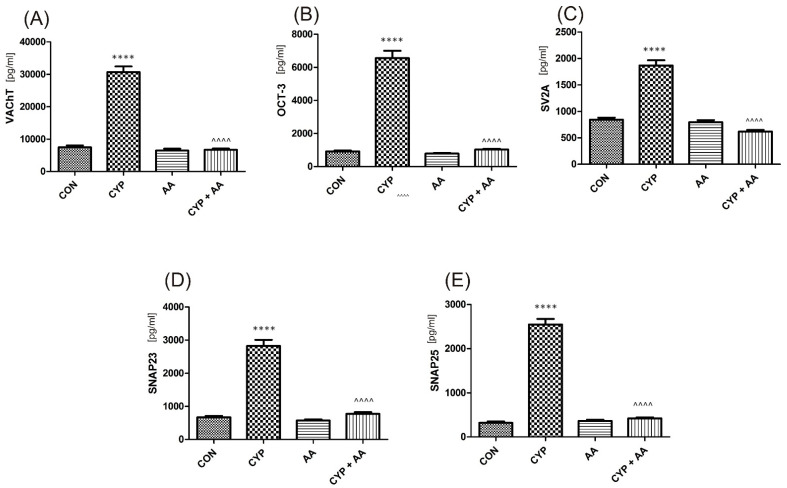
The effect of the two-week administration of asiatic acid (30 mg/kg/day for 14 days by oral gavage) on biomarkers in urothelium or in bladder detrusor muscle: (**A**) vesicular acetylcholine transporter (VAChT), (**B**) organic cation transporter 3 (OCT3), (**C**) rat synaptic vesicle glycoprotein 2A (SV2A), (**D**) synaptosomal-associated protein 23 (SNAP-23), and (**E**) synaptosome associated protein 25 (SNAP-25) in animals that were administered with a single injection of cyclophosphamide (CYP, 200 mg/kg, intraperitoneally). All results are presented as the mean ± SEM (*n* = 15 rats per group). The obtained data were assessed by the one-way ANOVA followed by Tukey’s post hoc test. * when significantly different from the control group. ^ when significantly different from the CYP group. **** or ^^^^ *p* < 0.0001. One-way ANOVA: for VAChT F(3.56) = 141, *p* < 0.0001, for OCT3 F(3.56) = 160, *p* < 0.0001, for SV2A F(3.56) = 95, *p* < 0.0001, for SNAP-23 F(3.56) = 116, *p* < 0.0001, and for SNAP-25 F(3.56) = 269, *p* < 0.0001. AA: asiatic acid; CON: control; CYP: cyclophosphamide.

**Figure 4:**
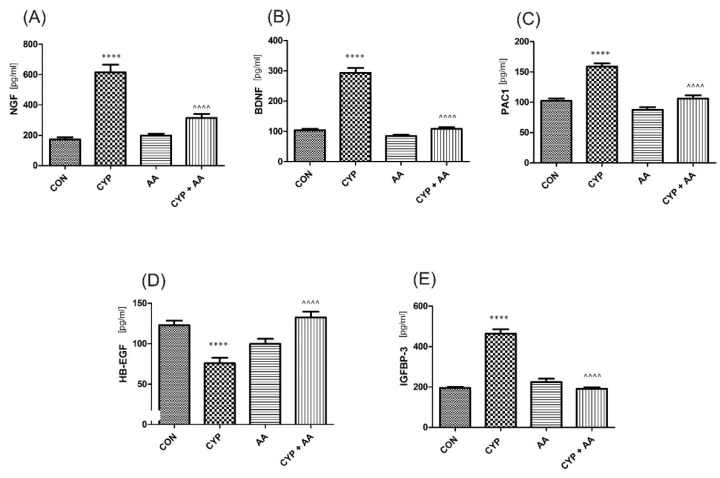
The effect of the two-week administration of asiatic acid (30 mg/kg/day for 14 days by oral gavage) on biomarkers in urothelium or in bladder detrusor muscle: (**A**) nerve growth factor (NGF), (**B**) brain-derived neurotrophic factor (BDNF), (**C**) PAC1 receptor (PAC1), (**D**) heparin-binding epidermal growth factor-like growth factor (HB-EGF), and (**E**) insulin-like growth factor-binding protein 3 (IGFBP-3) in animals that were administered with a single injection of cyclophosphamide (CYP, 200 mg/kg, intraperitoneally). All results are presented as the mean ± SEM (n = 15 rats per group). The obtained data were assessed by the one-way ANOVA followed by Tukey’s post hoc test. * when significantly different from the control group. ^ when significantly different from the CYP group. **** or ^^^^ *p* < 0.0001. One-way ANOVA: for NGF F(3.56) = 45, *p* < 0.0001, for BDNF F(3.56) = 113, *p* < 0.0001, for PAC1 F(3.56) = 41, *p* < 0.0001, for HB-EGF F(3.56) = 16, *p* < 0.0001, and for IGFBP-3 F(3.56) = 89, *p* < 0.0001. AA: asiatic acid; CON: control; CYP: cyclophosphamide.

There were no significant changes in the values of the bladder urothelium/detrusor muscle biomarkers after the administration of asiatic acid, when compared to animals from the saline-treated group (Figure 1, Figure 2, Figure 3 and Figure 4). Furthermore, in the cyclophosphamide-induced cystitis group (CYP group), concentrations of several markers were either elevated (BDNF, CGRP, OCT-3, VAChT, IL-1β, IL-6, NGF, nitrotyrosine, malondialdehyde, TNF-α, Rho kinase, SV2A, SNAP23, SNAP25, PAC-1, ORM1, occluding, and IGFBP-3) or decreased (HB-EGF, T–H protein, Z01, and HPX). As presented in Figure 1, Figure 2, Figure 3 and Figure 4 the administration of asiatic acid successfully restored concentrations of biomarkers in bladder urothelium, which was indicative of curative properties. Finally, the level of VAChT and Rho kinase in bladder detrusor muscle in CYP-injected animals after the application of asiatic acid was normalized (Figure 2 and Figure 3).

### 2.3. The Effects of Pre-Treatment with Asiatic Acid on Cyclophosphamide-Induced Changes in Urothelium Thickness

**Figure 5:**
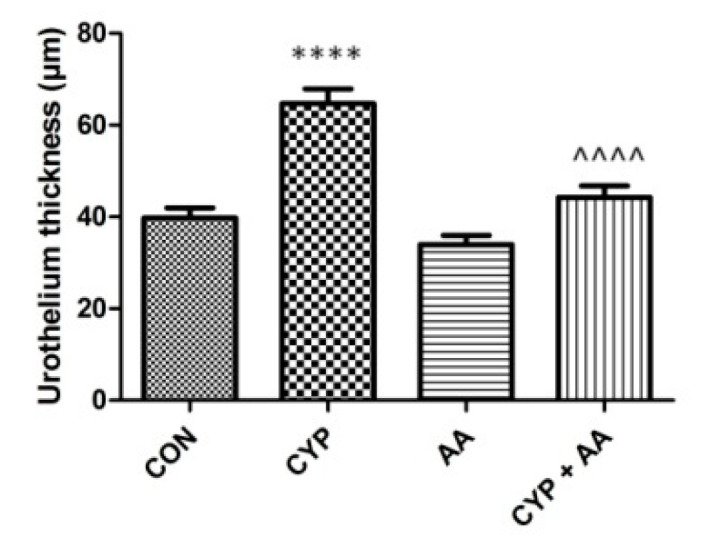
The effects of pretreatment with asiatic acid (30 mg/kg/day for 14 days by oral gavage) on cyclophosphamide (CYP, 200 mg/kg, i.p., single dose) induced changes in urothelium thickness. All results are presented as the mean ± SEM (n = 15 rats per group). The obtained data was assessed by the one-way ANOVA followed by Tukey’s post hoc test. * when significantly different from the control group. ^ when significantly different from the CYP group. **** or ^^^^ *p* < 0.0001. One-way ANOVA for urothelium thickness: F(3.56) = 29, *p* < 0.0001. AA: asiatic acid; CON: control; CYP: cyclophosphamide.

In the CYP group (Figure 5), an increase in bladder epithelial thickness was observed, when compared to the control group. No changes in the parameters were observed in the AA group. Finally, the administration of the asiatic acid to rats that received CYP significantly decreased urothelium thickness, compared to the CYP group.

### 2.4. The Results of Pretreatment with Asiatic Acid on Cyclophosphamide-Induced Changes in Bladder Oedema

**Figure 6:**
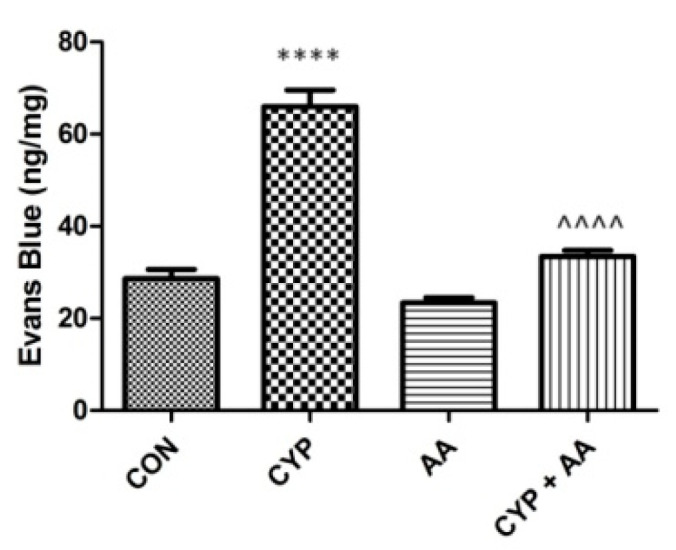
The effects of pretreatment with asiatic acid (30 mg/kg/day for 14 days by oral gavage) on cyclophosphamide (CYP, 200 mg/kg, i.p., single dose) induced changes in Evans Blue extravasation into bladder tissue. All results are presented as the mean ± SEM (n = 15 rats per group). The obtained data was assessed by the one-way ANOVA followed by Tukey’s post hoc test. * when significantly different from the control group. ^ when significantly different from the CYP group. **** or ^^^^ *p* < 0.0001. One-way ANOVA for Evans Blue extravasation: F(3.56) = 72, *p* < 0.0001. AA: asiatic acid; CON: control; CYP: cyclophosphamide.

The administration of cyclophosphamide resulted in significant changes in Evans Blue extravasation into the bladder tissue (Figure 6). Asiatic acid did not reveal a similar effect. However, in the CYP plus AA group Evans Blue extravasation was lowered markedly, when collated to the CYP group.

## 3. Discussion

Owing to its safe side effect profile and variety of molecular actions, AA has been introduced in the treatment of different diseases, but its influence on CYP-induced cystitis has not been determined so far. In our present study the protective efficacy of 14-day application via oral gavage at 30 mg/kg/day due to CYP-induced cystitis was analyzed. We showed for the first time that AA exhibits its bladder-protective influence through its antioxidant and anti-inflammatory effects due to the biochemical changes in both detrusor muscle and bladder urothelium. Furthermore, we presented that there was a notable influence on bladder oedema and urothelial permeability and respective urodynamic parameters.

In the current study, we were particularly interested in investigating the cystometric parameters that were deteriorated by the CYP and restored due to the administration of AA. In general, in CYP-induced cystitis animal models it is reduced bladder capacity and increased urinary frequency that are usually emphasized [17]. We showed that cyclophosphamide increased several parameters indicative of detrusor overactivity, i.e., DOI, ANVC, and FNVC, together with an elevation of BP and AUC. That was consistent with a decrease in VV, ICI, and BC. In our previous study on the retinyl acetate-induced model of detrusor overactivity, asiatic acid was effective in the normalization of mainly BC, DOI, ICI, TP, and VTNVC [18]. Thus, the main goal was to learn if these changes could be successfully restored after the administration of AA in a CYP model. We proved that the above-mentioned parameters returned to normal or close to normal values following constant application of the drug together with CYP. Furthermore, no changes in cystometric parameters, both characteristic of the filling and voiding phase, were observed in animals that received AA only. Decreased DOI seems to be a significant observation, as this parameter is a characteristic feature of an overactive bladder, indicative of the disease severity [18]. It is claimed that DOI represents the intensity of bladder contractions in a far more precise way compared to non-voiding contraction amplitude, non-voiding contraction frequency, and bladder compliance [19]. In the paper, due to AA administration we also observed an increased volume of bladder capacity that initiated bladder contraction. It is generally perceived as a proof of the effective treatment of an overactive bladder, because the increase itself leads to the reduction of micturition frequency and the number of episodes of urge urine incontinence [10].

It is thought that among serious consequences of the inflammatory response one cannot omit tissue damage apart from just malfunction [7]. IL 1β is a pivotal cytokine that triggers the immune response and stimulates the increase in the levels of other pro-inflammatory cytokines, i.e., TNF α and IL-6. The latter regulates the release of chemotactic factors and cell adhesion molecules [20,21]. As a consequence, authors pinpoint the presence of IL-1β, IL-6, and TNF-α together with malondialdehyde and 3-nitrotyrosineas indicators and mediators of inflammation [22]. In a paper by Smaldone et al., the authors claimed that increased urinary levels of IL-1 β and IL-6 were observed in cases with CYP-induced cystitis, while the value of its elevation correlated positively with the severity of the symptoms [23]. In the paper by Gomes et al. [24], administration of whole anti-IL- 1 β or anti-TNF-α serum in CYP-induced cystitis led to a significant decrease of the following parameters: mucosal erosion, hemorrhage, edema, and ulcerations. In our experiments, concentrations of several inflammatory markers were elevated (IL-1β, IL-6, NGF, nitrotyrosine, malondialdehyde, TNF-α) as well. However, the administration of AA normalized concentrations of these biomarkers in rat bladder urothelium, which meant a 1.4–3.8-fold decrease of the respective values when compared to the CYP group as presented in Figure 1. Another example of herbal medicine to be studied and already reported to have activity in models of CYP-induced cystitis is thymoquinone [25]. This compound ceased the delivery of pro-inflammatory molecules (i.e., IL-1, IL-6, and TNF-α), which in turn secured bladder cytoarchitecture and reduced immune cell infiltration within it. Furthermore, proteoglycans e.g., Tamm–Horsfall protein, are thought to be a crucial element of the lower urinary tracts’ defense, which is active in all inflammatory conditions with the elevated markers mentioned above [19]. In our experiments AA proved to be an anti-inflammatory agent by the elevation of T–H protein levels that were lowered in the CYP group.

Oxidative stress and chronic low-grade inflammation may promote a formation of by-products such as malondialdehyde during the process of lipid peroxidation [26]. Consistent with our studies, in the paper by Yuan et al. [27], a decrease in malondialdehyde concentration was seen after AA administration. Furthermore, it is thought that CYP generates oxidative stress mainly via inhibition of antioxidant enzymes [25]. Several neuropeptides, such as CGRP or substance P play a distinct role in the bladder dysfunction due to CYP [28]. Gonzales et al. found that CYP led to the accumulation of substance P and CGRP in the urinary bladder, indicative of oxidative stress [22]. In our study we observed a significant decrease in CGRP in AA-treated animals (755 ± 48 in the CYP group and 163 ± 8.2 in the CYP plus AA group). Finally, glycoprotein ORM1 is an acute phase protein that is elevated in inflammation [29], while HPX prevents heme-mediated oxidative stress [30]. In the present paper ORM1 levels were normalized and HPX values were restored after administration of AA in animals with CYP-induced cystitis.

Growth factors involved in regulation of micturition, i.e., NGF and BDNF, play an important role in the process of neuroinflammation [31]. A significant reduction of both factors was seen in our experiments after the application of AA (group CYP + AA). BDNF may be induced due to nerve damage and participates in chronic pain onset [32], while some authors claim it may become a biomarker of an overactive bladder [27]. In a paper by Ding et al., it acted via promotion of TNF-α and IL-1β release [31].

PAC 1 receptor exhibits tissue-specific distributions in the lower urinary tract [33]. Inhibition of PAC1 receptor decreased voiding frequency in animal models [34], while intravesical administration of the PAC1 receptor antagonist increased bladder capacity, and decreased the number of non-voiding bladder contractions in animals that received CYP [35]. In our experiments there was an observed normalization of PAC-1 concentration after AA administration in CYP-treated animals.

Some authors claim that Rho kinase antagonists may inhibit symptoms correlated with detrusor overactivity and induce anti-inflammatory reactions [36]. In our previous paper [37], the inhibition of Rho kinase resulted in a normalization of detrusor function and inflammatory activity. Here, we observed that Rho kinase concentration returned to a nearly normal value after treatment with AA (5587 ± 344 vs. 1749 ± 94).

Among CYP-induced changes, one mentions increased permeability of urothelium due to lowered concentration of ZO-1 and occludin [38]. ZO1 is a link between tight junction proteins and cytoskeleton, playing an important role in the paracellular barrier as well [39]. As a result, we aimed at a restoration of ZO-1 and occludin to the levels observed in saline-received animals. As displayed in Figure 2, there was a statistically significant increase in the levels of these biomarkers in the group treated with CYP plus AA, when compared with the CYP group.

Furthermore, there was a significant change in molecules that play a role in acetylcholine transportation [40]: VAChT and OCT3, or its release [41]: SNAP-23, SNAP-25, SVA2, as presented in Figure 3. Those findings were consistent with our previous studies [14,16]. Some authors pinpoint that the phenomena may lead to alterations in the autonomic nervous system [11], while others suggest that they may result in a contractile response in the rat urinary bladder [42].

HB-EGF is a growth factor that is engaged in the wound healing at the stages of e.g., epithelialization and wound contraction [43]. Keay et al. found that HB-EGF was significantly lowered in the urine of individuals with interstitial cystitis [44]. In our study, the same observation was reported in the CYP group, while a reverse change was found after the administration of AA. On the contrary, IGFBP3 is thought to be increased in patients with interstitial cystitis [43,44], and in our paper CYP-induced similar changes in bladder urothelium, ameliorated by AA.

Finally, in light of previous studies [14,16] and the present study, potential beneficial effects are to be observed in animals with active inflammatory processes and engaged oxidative stress than in controls. In the present study, there were no significant changes in the amounts of all the bladder biomarkers after the application of asiatic acid in monotherapy, when collated to rats in the control group.

As described above, CYP-induced cystitis involves oxidative stress and several inflammatory cascades. As a consequence, CYP-treated rats show clear signs of bladder wall thickening, including submucosal edema [45] and disorganization of the smooth muscle layer [46]. What is more, the authors claim that other abnormalities observed are mucosal detachment or necrosis in animal models due to CYP administration [47]. Our observations are in line with these publications. Worth emphasizing is the fact, however, that due to the efficient action of asiatic acid in rats that received CYP, a significantly decreased urothelial thickness was noticed. Last but not least is the influence of AA on the diminishing of bladder oedema (a significant decrease in Evans Blue extravasation). Finally, no toxic effects of AA were observed in the current and our previous studies [16] focused on AA in animal models.

There are some points to consider as potential limitations of the study. Some authors indicate the natural gender differences between male and female rat micturition [48], though as previously in our experiments e.g., [14,16] we focused on female rats. On the other hand, in the paper by Streng et al. no differences between the sexes as for the bladder pressure oscillations or in non-oscillatory voiding were found [48]. All the effects of AA presented in the paper should also be reassessed in clinical studies, as not all parameters used in animal conscious cystometry can be generalized to people. Finally, even though we received very promising data, some already developed derivatives of AA can become even more attractive for their potential use in the treatment of CYP-induced cystitis [5].

## 4. Materials and Methods

### 4.1. Animals

Sixty female Wistar rats (originally weighing 200–225 g, 10–12 weeks old) were used in the experiments. Rats were placed individually in the metabolic cages (3700M071, Tecniplast, West Chester, PA, USA) in environmentally controlled rooms (temperature of 22–23 °C, natural light/dark cycle, relative humidity ca. 45–55%) with unlimited access to food and water. The tested rats were randomly allocated to the four following experimental groups of 15 animals each:

1. Control (CON) group that received a single injection of vehicle I plus vehicle II for 14 days (CON group).
2. Cyclophosphamide (CYP) group that received a single injection of CYP (200 mg/kg) plus vehicle II for 14 days (CYP group).
3. Asiatic acid (AA) group that received a single injection of vehicle I plus asiatic acid (30 mg/kg/day for 14 days) (AA group).
4. CYP and AA group that received a single injection of CYP (200 mg/kg) plus asiatic acid (30 mg/kg/day for 14 days) (CYP + AA group).

### 4.2. Drugs

In the experiments, the following drugs were administered to the animals:

Cyclophosphamide (CYP; Endoxan, Baxter Deutschland GmbH, Unterschleiβheim, Germany), asiatic acid (AA; trans-(1R, 9S)-8-Methylene-4,11,11-trimethylbicyclo (7.2.0) undec-ene; Catalogue No.: BP0203, purity: 98%, company: Biopurify Phytochemicals Ltd., Chengdu, Sichuan, China).

CYP was diluted in a physiological saline (0.9% NaCl) and administered intraperitoneally (i.p.) as a single dose of 200 mg/kg, as described elsewhere [14,37]. Then, a 14-day treatment with asiatic acid (30 mg/kg/day) was started [4,16]. All the animals were weighed just before administration of the drug and received the tested substance in the respective dosage e.g., 200 mg/kg for CYP and 30 mg/kg for AA, but calculated for the actual weight of the rat. Asiatic acid was dissolved in 1% DMSO solution and it was administered by oral gavage. Oral, intraperitoneal, and intravenous routes were studied most in the majority of models [4]. The oral route is especially crucial for the future development of the drug for clinical practice. The available preclinical and clinical pharmacokinetic data pinpoint that AA is bioavailable in nearly all tissues after oral or interaperitoneal administration [4]. The control animals were given a volume-matched dose of the vehicles, i.e., vehicle I—an intraperitoneal injection of physiological saline (0.9% NaCl) and/or vehicle II—1% DMSO solution via oral gavage. Both doses of the injected drugs and the treatment schedules were chosen on the basis of our previous projects [16,17] and they were confirmed in preliminary studies carried out by our team. Cystometric studies and measurements of bladder oedema, urothelium thickness and biochemical analyses were performed 3 days after the last dose of asiatic acid.

### 4.3. Surgical Procedures

All the surgical procedures were performed as previously described [37], under anesthesia with i.p. injection of 75 mg/kg of ketamine hydrochloride (Ketanest; Pfizer, Warsaw, Poland) and 15 mg/kg of xylazine (Sedazin; Biowet, Puławy, Poland). The animals were injected subcutaneously with 100 mg of cefazolin sodium hydrate (Biofazolin; Sandoz, Warsaw, Poland) to prevent a urinary tract infection. The adequately prepared abdominal wall was opened through a vertical midline incision so as to make an easy access to the urinary bladder. After the necessary dissection of the bladder, a double lumen polyethylene catheter (BD, Franklin Lakes, NJ, USA), filled with physiological saline with a cuff at the end was inserted through a small opening of the bladder dome and attached with a 6-0 Vicryl suture. Finally, Healon (Pharmacia A.B., Warsaw, Poland) was used locally for anti-adhesive purposes. The abdomen was sutured in multiple layers.

### 4.4. Conscious Cystometry

Cystometric studies were carried out after 17 days after the surgical procedures (i.e., 3 days after the last dose of the asiatic acid), as we described before [14,37]. Briefly, the bladder catheter was linked through a three-way stopcock to a pressure transducer (FT03; Grass Instruments) placed at the level of the bladder and to a microinjection pump (CMA 100; Microject, Solna, Sweden) for documenting intravesical pressure and for administrating physiological saline into the bladder. The bladder was physiologically filled with saline at a constant rate of 0.05 mL/min, i.e., 3 mL/h at room temperature (22 °C) to obtain repetitive voiding based on our previous studies [14]. The analog signal from the transducer was amplified and digitized using the Polyview system (Grass Instruments, Herlev, Denmark). Micturition amounts were measured using a fluid collector attached to a force displacement transducer (FT03C; Grass Instruments). Both transducers were connected to a polygraph (7 DAG; Grass Instruments). Cystometry profiles and micturition volumes were recorded continuously on a Grass polygraph (Model 7E; Grass Instruments). The values measured in each rat represent the average of five bladder micturition cycles after obtaining repetitive voiding. The mean calculations from all animals in each condition were averaged to create pooled data for each condition. All procedures were performed by a person blinded to the treatments.

### 4.5. Biochemical Analyses

The levels of the following biomarkers were analyzed in the bladder urothelium using ELISA kits: brain-derived neurotrophic factor (BDNF; PROMEGA, CN G7610), calcitonin gene related peptide (CGRP; Biomatik, CN EKU02858), organic cation transporter 3 (OCT3; antibodies-online, CN ABIN6227163), heparin-binding epidermal growth factor-like growth factor (HB-EGF; Biomatik; CN EKU04742), interleukin 1-β (IL-1β; Cloud-Clone, CN SEA563Ra), interleukin 6 (IL-6; LifeSpanBioSciences; CN LS-F25921-1), nerve growth factor (NGF; LifeSpanBioSciences, CN LS-F25946-1), 3-nitrotyrosine (NIT; LifeSpanBioSciences; CN LS-F40120-1), malondialdehyde (MAL; Biomatik, CN EKF57996), Tamm–Horsfall protein (T-H protein, uromodulin; Antibodies-online; CN ABIN855058), tumor necrosis factor alpha (TNF-α; LifeSpanBioSciences, CN LS-F5193), tight junction protein 1 (ZO1; CUSABIO, CSB-E17287r), synaptosomal-associated protein 23 (SNAP-23; MyBioSource, MBS9317604), synaptosome associated protein 25 (SNAP-25; Biomatik, EKF58391), SV2A (rat synaptic vesicle glycoprotein 2A; MyBioSource; MBS9348576), PAC1 receptor (PAC1; LifeSpanBioSciences, LS-F17843), orosomucoid-1 (ORM1; MyBioSource.com), hemopexin (HPX; MyBioSource.com), occludin (OCC; MyBioSource.com), and insulin-like growth factor-binding protein 3 (IGFBP-3; MyBioSource.com). The level of Rho kinase (ROCK1; LifeSpanBioSciences, LS-F32208) and vesicular acetylcholine transporter (VAChT; LifeSpanBioSciences, CN LS-F12924-1) were determined in the bladder detrusor muscle. All measurements were carried out according to the manufacturers’ instructions. Each sample was measured in duplicate. The results are presented in pg/mL.

### 4.6. The Assessment of Urothelium Thickness

The urothelium thickness measurement was determined by the assessment of hematoxylin and eosin-stained sections in the manner previously described [37]. In brief, the Leica Qwin 500 Image Analyzer (Leica Imaging Systems Ltd., Cambridge, UK) was used to evaluate this parameter. Bladder epithelial thickness was assessed in low magnification (×10) and presented in micrometer. The presented values are the mean of 15 readings from 5 sections of each rat in a given study group.

### 4.7. The Assessment of Bladder Oedema

The measurement of bladder oedema was conducted by assessing vesical vascular permeability with the Evans Blue dye leakage technique, as previously described [14,37]. In brief, rats were injected with Evans Blue at a single dose of 50 mg/kg intravenously via a catheter located in the right femoral vein. After 30 min, the animals were sacrificed, bladders were excised and weighed, which were then cut lengthwise and placed in a volume of 1 mL of formamide solution at 56 °C for 24 h. Formamide absorbance analysis was performed at 620 nM comparing it to a standard curve. The obtained results were expressed in nanograms of Evans Blue per milligram of the urinary bladder.

### 4.8. Statistical Analysis

The obtained data was assessed by the one-way analysis of variance (ANOVA) followed by a Tukey post hoc test (Statistica, v. 10, StatSoft, Inc., Tulsa, OK, USA). All results are presented as the mean ± standard error of the mean (SEM). *p* < 0.05 was considered as statistically significant with 95% confidence.

## 5. Conclusions

In conclusion, asiatic acid was found to be an effective drug in the rat model of CYP-induced cystitis, reversing not only the inflammatory processes, but also being effective in the restoration of functional properties of the lower urinary tracts. Our experiments shed light on future clinical trials introducing the compound into the medial practice for the treatment of cyclophosphamide serious side effects.

## Figures and Tables

**Table 1:** The results of pretreatment with asiatic acid (AA) on cyclophosphamide-induced changes in cystometric parameters.

	BP cm H_2_O	TP cm H_2_O	MVP cm H_2_O	VV mL	PVR mL	ICI s	BC mL/cm H_2_O	DOI cm H_2_O/mL	A NVC cm H_2_O	F NVC Times/Filling Phase	VT NVC %	AUC cm H_2_O/s
**CON**	2.6 ± 0.19	6.3 ± 0.39	44 ± 1.7	0.96 ± 0.049	0.059 ± 0.0049	1061 ± 45	0.25 ± 0.014	53 ± 4.9	2.3 ± 0.099	0.36 ± 0.047	70 ± 3.1	14 ± 0.66
**CYP**	4.6 ± 0.21 ***	4.4 ± 0.21 **	37 ± 2.0	0.62 ± 0.043 *	0.053 ± 0.0047	731 ± 34 ***	0.16 ± 0.0091 ***	210 ± 16 ***	6.0 ± 0.29 ***	5.3 ± 0.32 ***	47 ± 3.3 **	22 ± 0.89 ***
**AA**	2.3 ± 0.13	6.4 ± 0.33	47 ± 3.3	0.88 ± 0.087	0.074 ± 0.0047	956 ± 35	0.23 ± 0.015	54 ± 6.7	2.5 ± 0.12	0.31 ± 0.025	64 ± 4.1	13 ± 0.76
**CYP** **+** **AA**	3.2 ± 0.22 ^^^	7.2 ± 0.37 ^^^	39 ± 2.9	0.94 ± 0.066 ^	0.070 ± 0.0049	999 ± 50 ^^	0.22 ± 0.0012 ^	92 ± 6.4 ^^^	3.0 ± 0.18 ^^^	1.2 ± 0.22 ^^^	66 ± 4.3 ^	15 ± 0.70 ^^^

Values are expressed as the mean ± SEM. *** *p* < 0.0001, ** *p* < 0.001, * *p* < 0.05 when significantly different from the control group; ^^^ *p* < 0.0001, ^^ *p* < 0.001, ^ *p* < 0.05 when significantly different from the CYP group. One-way ANOVA: for BP F(3.56) = 1.7, *p* < 0.0001, for TP F(3.56) = 13, *p* < 0.0001, for MVP F(3.56) = 3.4, *p* < 0.05, for VV F(3.56) = 6.3, *p* < 0.0001, for PVR F(3.56) = 3.9, *p* < 0.05, for ICI F(3.56) = 12, *p* < 0.0001, for BC F(3.56) = 9.7, *p* < 0.0001, for DOI F(3.56) = 61, *p* < 0.0001, for ANVC F(3.56) = 85, *p* < 0.0001, for FNVC F(3.56) = 144, *p* < 0.0001, for VTNVC F(3.56) = 7.7, *p* < 0.001, and for AUC F(3.56) = 26, *p* < 0.0001. BP: basal pressure (cm H_2_O), TP: threshold pressure (cm H_2_O), MVP: micturition voiding pressure (cm H_2_O), VV: voided volume (mL), PVR: post-void residual (mL), ICI: intercontraction interval(s), BC: bladder compliance (mL/cm H_2_O), DOI:detrusor overactivity index (cm H_2_O/mL), ANVC: non-voiding contraction amplitude (cm H_2_O), FNVC: non-voiding contraction frequency (times/filling phase), VTNVC: volume threshold to elicit NVC (%), AUC: the area under the pressure curve (cm H_2_O/s). AA: asiatic acid; CON: control; CYP: cyclophosphamide.

## Data Availability

Data is contained within the article.

## Abbreviations

ANVC	non-voiding contraction amplitude (cm H_2_O)
AUC	the area under the pressure curve (cm H_2_O/s)
BC	bladder compliance (mL/cm H_2_O)
BDNF	brain-derived neurotrophic factor (pg/mL)
BP	basal pressure (cm H_2_O)
CGRP	calcitonin gene related peptide (pg/mL)
DO	detrusor overactivity
DOI	detrusor overactivity index (cm H_2_O/mL)
FNVC	non-voiding contraction frequency (times/filling phase)
HB-EGF	heparin-binding EGF-like growth factor (pg/mL)
HPX	hemopexin (pg/mL)
ICI	intercontraction interval (s)
IGFBP-3	insulin-like growth factor-binding protein 3 (pg/mL)
IL-1β	interleukin 1-β (pg/mL)
IL-6	interleukin 6 (pg/mL)
MAL	malondialdehyde (pg/mL)
MVP	micturition voiding pressure (cm H_2_O)
NGF	nerve growth factor (pg/mL)
NIT	3-nitrotyrosine (pg/mL)
OAB	overactive bladder syndrome
OCC	occludin (pg/mL)
OCT3	organic cation transporter 3 (pg/mL)
ORM1	orosomucoid-1 (pg/mL)
PAC1	PAC1 receptor (pg/mL)
PVR	post-void residual (mL)
ROCK1	Rho kinase (pg/mL)
SNAP-23	synaptosomal-associated protein 23 (pg/mL)
SNAP-25	synaptosome associated protein 25 (pg/mL)
SV2A	rat Synaptic vesicle glycoprotein 2A (pg/mL)
T–H	Tamm–Horsfall protein (pg/mL)
TNF-α	tumor necrosis factor alpha (pg/mL)
TP	threshold pressure (cm H_2_O)
UT	urothelium thickness (am)
VAChT	vesicular acetylcholine transporter (pg/mL)
VTNVC	volume threshold to elicit NVC (%)
VV	voided volume (mL)
Z01	tight junction protein 1 (pg/mL)
